# Influence of Metabolic Parameters and Treatment Method on OCT Angiography Results in Children with Type 1 Diabetes

**DOI:** 10.1155/2020/4742952

**Published:** 2020-11-18

**Authors:** Marta Wysocka-Mincewicz, Marta Baszyńska-Wilk, Joanna Gołębiewska, Andrzej Olechowski, Aleksandra Byczyńska, Wojciech Hautz, Mieczysław Szalecki

**Affiliations:** ^1^Department of Endocrinology and Diabetology, The Children's Memorial Health Institute, Warsaw, Poland; ^2^Department of Ophthalmology, The Children's Memorial Health Institute, Warsaw, Poland; ^3^Lazarski University, Faculty of Medicine, Warsaw, Poland; ^4^Collegium Medicum, Jan Kochanowski University, Kielce, Poland

## Abstract

**Aim:**

To evaluate the influence of metabolic parameters and the treatment method in children with type 1 diabetes (T1D) on the optical coherence tomography angiography (OCTA) results as early markers of diabetic retinopathy (DR). *Material and Methods*. This prospective study enrolled 175 consecutive children with T1D. OCTA was performed using AngioVue (Avanti, Optovue). Whole superficial capillary vessel density (wsVD), fovea superficial vessel density (fsVD), parafovea superficial vessel density (psVD), whole deep vessel density (wdVD), fovea deep vessel density (fdVD), parafovea deep vessel density (pdVD), foveal thickness (FT), parafoveal thickness (PFT), and foveal avascular zone (FAZ) in superficial plexus were evaluated and analyzed in relation to individual characteristics, i.e., sex, weight, height, body mass index (BMI), and metabolic factors: current and mean value of glycated hemoglobin A1c (HbA1c). Furthermore, the analysis concerned the diabetes duration, age at the T1D onset, and type of treatment—multiple daily insulin injections (MDI) or continuous subcutaneous insulin infusion (CSII).

**Results:**

In the study group, we did not identify any patient with DR in fundus ophthalmoscopy. Age at the onset of diabetes correlated negatively with FAZ (*r* = −0.17, *p* < 0.05). The higher level of HbA1c corresponded to a decrease of wsVD (*r* = −0.13, *p* < 0.05). We found significantly lower fsVD (32.25 ± .1 vs. 33.98 ± .1, *p* < 0.01), wdVD (57.87 ± .1 vs. 58.64 ± .9, *p* < 0.01), and pdVD (60.60 ± .2 vs. 61.49 ± .1, *p* < 0.01) and larger FAZ area (0.25 ± .1 vs. 0.23 ± .1, *p* < 0.05) in the CSII vs. MDI group.

**Conclusion:**

The metabolic parameters, age of the onset of diabetes, and treatment method affected the OCTA results in children with T1D. Further studies and observation of these young patients are needed to determine if these findings are important for early detection of DR or predictive of future DR severity.

## 1. Introduction

Type 1 diabetes (T1D) is the third most common chronic disease in children. Diabetic retinopathy (DR) is the most common microvascular complication of diabetes, and it develops in most patients with long-standing T1D [1]. However, in pediatric population, DR is very rare. The pubertal status and the prepubertal duration of diabetes influence the risk of developing DR, as children under the age of 10 have minimal risk, and no cases of proliferative DR in the first decade of life were noted [2, 3]. New imaging technologies would be useful in the early identification of retinal structural and functional changes, before DR is clinically detectable. Optical coherence tomography angiography (OCTA) is a new, noninvasive tool, based on the detection and measurement of intravascular erythrocyte movement [4]. OCTA enables reproducible, quantitative assessment of the retinal microcirculation and seems to be an effective method in the detection of early microcirculation disorders. The aim of the study was to assess the influence of metabolic parameters and the treatment method on OCTA results in children with T1D.

## 2. Material and Methods

### 2.1. Patients

This prospective, observational study enrolled 175 consecutive Caucasian children with T1D remaining under control of the Department of Endocrinology and Diabetology of The Children's Memorial Health Institute, which met criteria and agreed to study participation. The study was approved by the Bioethics Committee of The Children's Memorial Health Institute in Warsaw and followed the tenets of the Declaration of Helsinki. Written informed consent was obtained from the patient's legal guardian and from patients > 16 years old after an explanation of the nature of the noninvasive study. The inclusion criteria were a diagnosis of T1D based on the International Society for Pediatric and Adolescent Diabetes (ISPAD) criteria and insulin treatment (not in full remission). In the study, 25 children have newly recognized T1D (from 2 weeks up to a year after the diagnosis). The exclusion criteria were the history of prematurity, other concomitant retinal pathologies, such as hereditary retinal dystrophies, vitreoretinal diseases, myopia or hypermetropia (more than 6 diopters), and history of uveitis.

Metabolic control was measured by the current glycated hemoglobin A1c level (HbA1c) and the mean value for the whole T1D duration (minimum 4 tests per year), the amount of insulin per kilogram of the weight, the mean total daily insulin (taken from the pump memory or profile led in hospital), and the dose of insulin for breakfast (as one of the markers connected with insulin resistance). In the study, the weight, the height, body mass index (BMI), BMI *Z*-score, the age at onset, and the T1D duration were evaluated.

The BMI *Z*-score was calculated using the LMS method based on the Box-Cox transformation [5, 6].

(1) $$\begin{array}{c} Z‐\text{score}\left(x\right)=\frac{{\left(X/M\right)}^{L}-1}{LxS}, \end{array}$$

where *X* is a measured anthropometric parameter (e.g., height and BMI), *M* is the median of the value, *L* is the power of the Box-Cox' transformacy, and *S* is a variability coefficient. *L*, *M*, and *S* values were taken from the reference tables for the chosen anthropometric parameters for the determined age and sex (by Kułaga et al.) [7, 8]. We checked the effect of the presence of diabetic ketoacidosis (DKA) at the time of the T1D onset. The next part of the analysis concerned the type of treatment—multiple daily insulin injections (MDI) or continuous subcutaneous insulin infusion (CSII) at the moment of taking the OCTA images. The MDI group included 81 children (the mean diabetes duration 3.38 ± 3.0 years) treated by pens in the basal-bolus functionally scheme, when the mean dose of the insulin analogue is calculated dependently on the amount of food and correction, or children with strict doses for the main meals. The CSII group consisted of 87 children treated with an insulin pump for more than a year (the mean duration of CSII 3.74 ± 1.9 years, the mean duration of T1D 5.47 ± 3.3 years), but not all of them strictly followed the recommendations of the diabetes care team (strict doses per meal, not weighed meals) (Table 1).

### 2.2. Database

OCTA was performed using a commercially available RTVue XR Avanti with AngioVue (Optovue, Fremont, CA, USA) with 3mm × 3mm images of macula, centered on the foveola. Each OCTA en face image contains 304 × 304 pixels created from the intersection of the 304 vertical and the 304 horizontal B-scans. AngioVue automatically segments the area into four layers, including superficial capillary plexus layer (SP), deep capillary plexus layer (DP), outer retinal layer, and choriocapillaris. The SP en face image was segmental with an inner boundary at 3 *μ*m beneath the internal limiting membrane and outer boundary set at 15 *μ*m, beneath the inner plexiform layer, whereas the deep capillary plexus en face image was segmented with an inner boundary at 15 *μ*m beneath the inner plexiform layer and an outer boundary at 70 *μ*m beneath the inner plexiform layer. Integrated automated algorithms provided by the machine software were used to quantify the foveal avascular zone (FAZ) (mm^2^) and macular vascular density (%). FAZ area was automatically calculated for superficial plexus; capillary vascular density in the macular and paramacular region was measured both in superficial and deep plexuses. Vessel density is calculated as the percentage area occupied by following blood vessels in the selecting region, which enables the quantitative assessment of microvasculature. The whole superficial capillary vessel density (wsVD), fovea superficial vessel density (fsVD), parafovea superficial vessel density (psVD), whole deep vessel density (wdVD), fovea deep vessel density (fdVD), and parafovea deep vessel density (pdVD) were taken into analysis. Foveal thickness (FT) (*μ*m) and parafoveal thickness (PFT) (*μ*m) data were obtained from retinal maps, using the same device. All subjects were dilated with 1% tropicamide eye drops before examination. The scans for each eye were captured; then, the best one in quality (with a signal strength index > 60) was considered for analysis. Two trained OCTA readers reviewed all images independently to ensure correct segmentation and identify poor quality scans, with motion artifacts or blurred images, where data were insufficient for proper analysis. The data of both eyes of patients were taken into all analysis independently, because of intraeye differences and metabolic parameters influenced on both eyes. After exclusion of eyes with poor quality scans, 330 eyes (from 168 patients) were taken to the final analysis.

### 2.3. Statistical Analysis

The data was described by mean, median, standard deviation, and minimum and maximum values. Values with normal distribution (which was checked using the Shapiro-Wilks test) were analyzed by Pearson correlation, but those which were not normal in distribution were analyzed by Spearman rank *R* correlations. The differences between the two groups were tested by the unpaired Student *t*-test or Mann-Whitney *U* test as appropriate. A level *p* < 0.05 was recognized as statistically significant. The tests were done using the Statistica 6.0 StatSoft Company.

## 3. Results

The characteristics of the study population are summarized in Table 1.

In the study group, we did not identify any patient with DR in fundus ophthalmoscopy.

We did not find any significant correlation between the T1D duration and the OCTA parameters. Age at the onset of T1D correlated significantly positively with FT (*r* = 0.12, *p* < 0.05) and negatively with FAZ (*r* = −0.17, *p* < 0.05).

**Figure 1:**
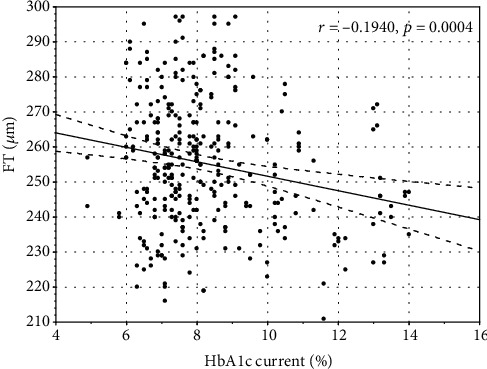
The correlation between current glycated hemoglobin A1c and foveal thickness.

**Figure 2:**
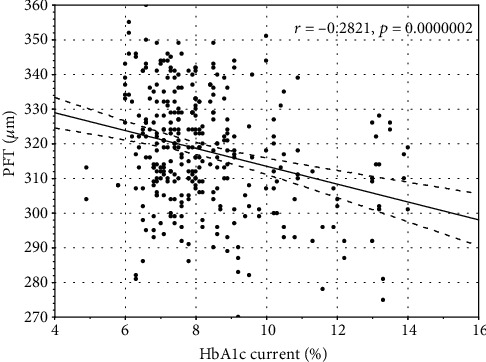
The correlation between current glycated hemoglobin A1c and parafoveal thickness.

**Figure 3:**
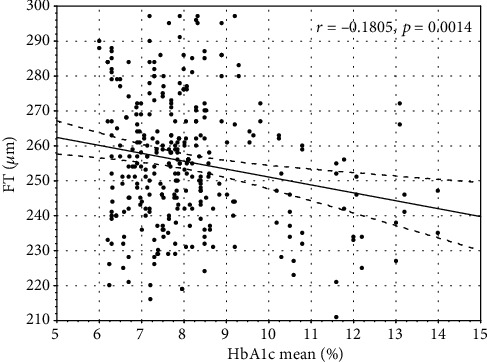
The correlation between mean average glycated hemoglobin A1c and foveal thickness.

**Figure 4:**
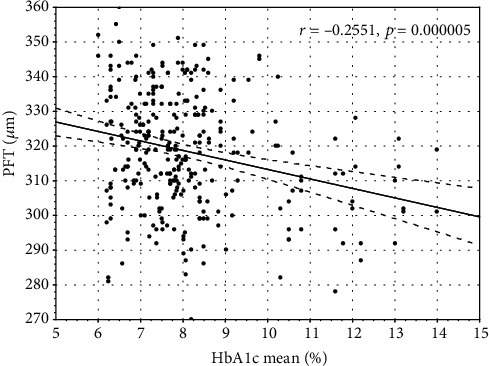
The correlation between mean average glycated hemoglobin A1c and parafoveal thickness.

Mean HbA1c correlated negatively with wsVD (*r* = −0.13, *p* < 0.05). HbA1c current and mean correlated with FT (*r* = −0.19, *p* ≤ 0.01 and *r* = −0.18, *p* < 0.02, respectively) and PFT (*r* = −0.28, *p* ≤ 0.01 and *r* = −0.26, *p* ≤ 0.01, respectively) (Figures 1–4).

The weight and the height influenced on FT (*r* = 0.13, *p* = 0.05 and *r* = 0.13, *p* < 0.05, respectively), however, no correlation between BMI or BMI *Z*-score and FT were found.

The influence of DKA at the onset of T1D measured by pH correlated with FT and PFT (*r* = 0.18, *p* = 0.02 and *r* = 0.20, *p* < 0.01, respectively), psVD (*r* = 0.18, *p* < 0.05), and fdVD (*r* = 0.20, *p* < 0.02).

When dividing the group by sex (83 girls, 86 boys), none of the parameters such T1D duration, age at T1D onset, weight, height, BMI *Z*-score, and current and mean HbA1c were statistically different. The groups were statistically different in OCTA parameters: FT (girls 250.40 ± 17.9 vs. boys 260.20 ± 19.2, *p* < 0.01), PFT (314.97 ± 17.1 vs. 321.25 ± 15.3, *p* < 0.01), fsVD (32.39 ± 5.1 vs. 33.87 ± 5.2, *p* < 0.05, respectively), fdVD (31.52 ± 6.1 vs. 33.43 ± 5.6, *p* < 0.01, respectively), and pdVD (61.30 ± 2.2 vs. 60.70 ± 2.2, *p* < 0.03, respectively).

Statistically significant correlations were found between the duration of CSII treatment and FT (*r* = 0.19, *p* < 0.01), FAZ (*r* = −0.17, *p* < 0.05), fsVD (*r* = 0.20, *p* < 0.01). The dose of insulin per day positively correlated only with FT (*r* = 0.12, *p* < 0.05). There were no significant correlations between the OCTA parameters and the amount of basal insulin or the amount of insulin per breakfast.

After dividing the groups by the type of treatment (MDI = 81 or CSII = 87), we recorded the differences between the groups regarding the T1D duration, age at T1D onset, weight, height, and mean HbA1c (Table 2). These groups were statistically different in FAZ, fsVD, wdVD, and pdVD but not in FT and PFT.

## 4. Discussion

Nowadays, we do not observe clinical signs of diabetic retinopathy in pediatric population, as in this study group was not any child with this complication. Similarly, among 370 children with DM1 enrolled in the study of Geloneck et al., no patient was diagnosed with DR [9]. Probably, this results from better metabolic control effect, more physiological treatment using CSII by means of an insulin pump, more frequent use of continuous glucose monitoring, and more active and aggressive treatment based on monitoring trends. However, the analysis from the United Kingdom demonstrated that 9.5% of children under 12 years old had signs of background DR [10]. Our study also included children with poor metabolic control, but none of them had any signs of DR (neither in fundoscopy nor on fundus color photography), even after 14 years of T1D duration. We performed OCTA to analyze possible early structural and/or functional dependencies with clinical parameters in pediatric population. Early DR is clinically diagnosed by observing microaneurysms using fundoscopy. Preclinical DR may be accompanied by initial vascular abnormalities of the capillaries before the occurrence of microaneurysms.

Pediatric patients are the most important population of T1D, because of long life duration of this disease and its influence on all tissues. We detected weak but very significant negative correlations between the OCTA parameters and the parameters of diabetic metabolic control. In our previous studies, we did not observe such dependencies possibly because of smaller homogeneity of the study group [11].

Analyzing the groups undergoing different methods of treatment, we showed significantly lower fsVD, wdVD, and pdVD and a larger FAZ area in the CSII group. These results were completely surprising because we expected better retinal perfusion in children on CSII therapy, which is a more physiological type of treatment. But this group had longer T1D duration and an earlier T1D onset, which could partly justify these findings; however, T1D duration did not correlate with any OCTA parameters, and age at T1D onset correlated only with FAZ and FT. These groups differed in many demographic and metabolic parameters, but the same variables had no influence on the OCTA data in the other analyses and correlations. The FAZ area is considered as one of the most important parameters in OCTA, which could help evaluate the risk of developing DR in diabetic patients without any retinal changes on clinical examination. To avoid the effect of axial length and age on FAZ, baseline characteristics were similar between the study groups; the groups were comparable in terms of age, gender, and refraction. Other authors have reported the same approach to minimize FAZ variability in their studies [12, 13].

In the study of Niestrata-Ortiz et al., the FAZ parameters were increased proportionally to the diabetes duration [14], which was not reflected in our analysis, similarly as in other authors' research [15]. Statistically larger FAZ in diabetic patients was noted by the other authors too [15–17], but not in our previous study [6] and Tam et al. [18].

When we analyzed the differences between girls and boys, the FAZ area was smaller in boys, but this difference was not statistically significant, which Niestrata-Ortiz et al. observed [19]. They revealed the smaller mean FAZ surface area in boys similarly in the diabetic and control groups, both in superficial and deep capillary plexuses. In our study, we detected significantly higher central foveal and parafoveal thickness in the group of boys. These results could confirm the physiological differences between the genders, regardless of T1D.

Another significant factor is the age at the onset of T1D. In our study, this parameter negatively correlated with FAZ. This is contrary to the research by Onoe et al., where the larger FAZ area was associated with the older onset age [20] or to the study of Mameli et al., in which the correlation was not statistically significant [21]. Nevertheless, the number of participants in the aforementioned studies was remarkably smaller in comparison with our analysis.

In our study, mean HbA1c correlated negatively with wsVD. Wang et al. revealed that for every 1-point increase in HbA1c, the hazard for DR increased by 20% among youths with T1D (hazardratio(HR) = 1.20; 95% confidence interval (CI) 1.06-1.35) [22]. Conversely, several studies indicate the lack of correlation between mean HbA1c and retinal vessel density in patients with T1D [17, 23]. Due to the discordance in literature, the authors suggest a greater focus on new parameters in clinical practice, i.e., “time in range” (described as the percentage of time when a patient has a blood glucose level in the target range) [17]. The value of this parameter has already been proven in type 2 diabetes [24]. We have to remember that such a parameter describes a short period of the patient's life—only this time when we have a monitoring memory. In the near future when almost the whole population will be subject to monitoring systems, this factor may play a crucial role in the process of unification and analysis of the same parameters.

In our study, we also performed the analysis of the parameters regarding the onset of T1D. DKA measured by pH at the moment of the diagnosis correlated with psVD, fdVD, FT, and PFT. To our knowledge, this is the first report on such dependence. In the literature, we found one study which analyzed the DKA status and its influence on the OCT parameters (OCT performed at the onset of T1D). Jeziorny et al. reported no significant difference in the retinal nerve fiber layer (RNFL) in OCT between DKA and non-DKA patients [25]. Currently, uncertainty remains with regard to the relationship between the severity of DKA at the moment of the diagnosis and the risk of DR. In one study of 230 patients with childhood-onset T1D, the authors (after 20 years of observation) did not confirm such a correlation [26].

## 5. Conclusion

To our knowledge, this is the biggest analysis of diabetic children, using OCT angiography, which considered the influence of many possible metabolic, demographic, and treatment parameters. In our study, we found significant correlations between, e.g., HbA1c, DKA, age at the onset of T1D, the treatment method, and the OCTA results in children with T1D. The detected correlations were predominantly weak, but statistically significant. All the children participated in the study had no changes suggested DR; probably in children, there is a strong capacity for autoregulation, and because of that, our correlations were so weak. Further studies are needed to detect the most important parameters for early detection of DR.

## Figures and Tables

**Table 1:** The characteristics of the studied patients.

Investigative trait	Mean	SD	Median	Min	Max
Age (years)	12.74	±3.7	13.00	4.50	18.00
Diabetes duration (years)	5.48	±3.7	4.46	0.02	15.33
Age at onset (years)	8.30	±3.8	7.98	1.46	17.04
Weight (kg)	47.70	±18.6	46.00	16.50	97.00
Height (cm)	155.40	±19.5	159.99	106.00	181.50
BMI (kg/m^2^)	18.94	±4.3	17.90	11.99	32.56
HbA1c current (%)	8.3	±1.8	7.9	5.8	14.0
HbA1c mean (%)	7.8	±1.2	7.7	6.0	12.0

SD = standard deviation; BMI = body mass index; HbA1c = glycated hemoglobin A1c.

**Table 2:** Comparison of the groups treated by MDI or CSII.

	MDI, *n* = 81 Mean ± SD	CSII, *n* = 87 Mean ± SD	*p* level
Age (years)	12.69 ± 3.8	12.91 ± 3.5	*p* = 0.58
Age at T1D onset (years)	9.10 ± 4.0	7.40 ± 3.5	*p* < 0.01
Diabetes duration (years)	3.40 ± 3.3	5.40 ± 3.4	*p* ≤ 0.01
Weight (kg)	45.40 ± 17.3	49.50 ± 19.4	*p* < 0.05
Height (cm)	152.80 ± 19.1	157.50 ± 19.7	*p* < 0.05
Mean HbA1c (%)	8.6 ± 1.8	7.7 ± 1.1	*p* ≤ 0.01
FT	255.20 ± 19.0	255.40 ± 19.0	*p* = 0.90
PFT	317.60 ± 17.0	318.80 ± 15.0	*p* = 0.50
FAZ	0.23 ± 0.1	0.25 ± 0.1	*p* < 0.05
wsVD	51.50 ± 3.0	51.10 ± 2.7	*p* = 0.30
fsVD	33.98 ± 5.1	32.25 ± 5.1	*p* < 0.01
psVD	53.04 ± 3.4	52.70 ± 2.9	*p* = 0.40
wdVD	58.64 ± 1.9	57.87 ± 2.1	*p* < 0.01
fdVD	33.08 ± 5.6	31.85 ± 6.2	*p* = 0.09
pdVD	61.49 ± 2.1	60.60 ± 2.2	*p* < 0.01

MDI = multiple daily insulin injections; CSII = continuous subcutaneous insulin infusion; T1D = type 1 diabetes; HbA1c = glycated hemoglobin A1c; wsVD = whole superficial capillary vessel density; fsVD = fovea superficial vessel density; psVD = parafovea superficial vessel density; wdVD = whole deep vessel density; fdVD = fovea deep vessel density; pdVD = parafovea deep vessel density; FAZ = foveal avascular zone.

## Data Availability

Due to the nature of this research, participants of this study did not agree for their data to be shared publicly, so supporting data is not available.
